# Anticancer activity of cow, sheep, goat, mare, donkey and camel milks and their caseins and whey proteins and in silico comparison of the caseins

**Published:** 2017-06

**Authors:** Malihe Shariatikia, Mandana Behbahani, Hassan Mohabatkar

**Affiliations:** Department of Biotechnology, Faculty of Advanced Sciences and Technologies, University of Isfahan, Isfahan, Iran

**Keywords:** Milks, Casein, Whey proteins, MTT assay, *In silico* study

## Abstract

The present investigation was carried out to evaluate anticancer activity of cow, goat, sheep, mare, donkey and camel milks and their casein and whey proteins against MCF7 cell line. The structure-based properties of the casein proteins were also investigated, using bioinformatics tools to find explanation for their antitumor activities. The effect of different milks and their casein and whey proteins on MCF7 proliferation was measured using MTT assay at different concentrations (0.5, 1 and 2 mg/ml). The results showed that mare, donkey, cow and camel milks and their casein and whey proteins have potent cytotoxic activity against MCF7 cells in a dose dependent manner while sheep and goat milks and their proteins did not reveal any cytotoxic activity. The *in silico* results demonstrated that mare, donkey and camel caseins had highest positive and negative charges. The secondary structure prediction indicated that mare and donkey caseins had the maximum percentage of α helix and camel casein had the highest percentage of extended strand. This study suggests that there is a striking correlation between anti-cancer activity of milk caseins and their physicochemical properties such as alpha helix structure and positive and negative charges. In conclusion, the results indicated that mare, camel and donkey milks might be good candidates against breast cancer cells.

## INTRODUCTION

Cancer is one of the most common malignancies worldwide. There fore, discovery and development of novel anticancer agents with new modes of action is greatly needed. Many researches reported that milk components possessed biological properties beyond their nutritional significance. Biological functions of milk are mainly due to milk peptides and proteins. Milk proteins include of approximately 20% whey and 80% casein. Whey contains five major proteins, including α-lactalbumin, glycomacropeptide, β-lactoglobulin, immunoglobulins and serum albumin. Casein contains αs1, αs2, β and κ casein [1]. There have been several reports of antibacterial, antiviral, antifungal and antioxidant activities of casein and whey proteins [2].

The immunomodulatory function of milk and casein peptides and whey proteins were also reported [3-5]. Several studies were also indicated that whey proteins such as lysozyme, lactoferrin and bovine serum albumin possess effective anti-tumor activities. Xueying Mao et al reported that whey proteins of donkey milk have potent anti-proliferative activity against lung cancer. Five different casomorphins; αs1-CN (f90–95), αs1-CN (f 90–96), β-casomorphin-7, β-casomorphin-7 (f1-5) and morphiceptin were also reported to inhibit cell proliferation of human breast cancer cell line [6, 7]. However, no scientific study has been presented yet about the activities of donkey, goat, sheep and mare milks against MCF7 cells. In the present study, casein and whey proteins of donkey, camel, sheep, goat, cow and mare milks were isolated and incubated with breast cancer cell lines (MCF7). The effect of six different milks (donkey, camel, sheep, goat, cow and mare) and their caseins and whey proteins on growth inhibition of MCF7 cells was investigated. In addition, in the present study different features of the casein were also investigated, using bioinformatics tools to find explanations for their antitumor activities.

## MATERIALS AND METHODS

**Milk processing:** Milk from different farms in Isfahan (Iran) was used in this study. Milk samples were collected and heated in a thermostatic water bath at pre-pasteurization temperature of 63ºC for 20 min and cooled to 4ºC. Samples were stored at -20ºC until analysis [6]. Skimmed milk was prepared from fresh milk by centrifugation at 5000 × *g* for 20 min at 4ºC and the fat layer was drawn up [5]. Whole casein of milk was obtained from skimmed milk by adjusting the pH to 4.6 (the Iso-electric point of casein), and centrifuged at 8000 × *g* for 20 min at 20ºC to obtain a supernatant of whey proteins [8]. Whey proteins were obtained after precipitation of caseins. Whey proteins were washed and centrifuged thrice, then their pH was adjusted to 6.8 using 1N NaOH [5]. The casein and whey proteins were then lyophilized and stored at -20°C [6].

**Cell culture and Cytotoxicity assay:** MCF-7 (human breast cancer) cell lines were purchased from National Cell Bank of Pasture Institute, Tehran, Iran. Cell lines were maintained in RPMI supplemented with 10% (v/v) heat-inactivated fetal bovine serum (FBS), 100 U ml^-1^ penicillin and 100 μg ml^-1^ streptomycin and 5 mM L- glutamine. The cell lines were maintained at 37ºC in a humidified incubator (N-Biotek Korea) containing 5% CO_2_ under *Mycoplasma*-free conditions [9]. 

The effect of different concentrations (0.5, 1 and 2 mg/ml) of milk and casein and whey proteins on MCF-7 cells was determined through a modified 3- (4, 5-dimethylthiazol-2-yl)-2, 5-diphenyl tetrazolium bromide (MTT) assay [10]. After 48 h of incubation at 37°C, MTT solution (5 mg/ml) was added to each well, and the plate was incubated for 4 h. Finally, 50 μl of PrOH/HCl/TX (0.04M HCl in 2-propanol plus 10% Triton X-100) was added to solubilize the formed formazan crystals [11]. The plate was re-incubated for 24 h and amount of formazan crystal was determin-ed by measuring the absorbance at 492 nm using a micro plate spectrophotometer (Awareness Technology Inc., stat fax 2100). All of material was purchased from Gibco Company (Germany). The percentage of survival cells was calculated as follows: Cell viability = ($\frac{A_{\mathrm{treated}}}{A_{\mathrm{control}}}$) ×100

**Retrieval of target amino acid sequences and Multiple Sequence Alignment:** Casein alpha S1 proteins from sheep, goat, cow, camel, horse and donkey were analyzed in the present study. The protein sequences are available at NCBI. The accession numbers and number of amino acids of αs1-Casein are shown in Table 1. The CLUSTAL Omega at European Bioinformatics Institute was used for generating a multiple sequence alignment [MSA] of casein alpha S1 protein sequences from six different species [12].

**Table 1:** Milk protein sequence entries in NCBI database and the number of amino acids

**Protein**	**Proteins accession numbers**	**Number of amino acids**
**αs1-Casein [** ***Bos taurus*** **]**	ABW98936.1	214
**αs1-Casein [** ***Equus caballus*** **]**	AAK83668.1	212
**αs1-Casein [** ***Equus asinus*** **]**	P86272.1	217
**αs1-Casein [** ***Camelus dromedarius*** **]**	CAA10077.1	222
**αs1-Casein [** ***Capra hircus*** **]**	CAD45345.1	214
**αs1-Casein [** ***Ovis aries*** **]**	AEN84772.1	214

**Phylogenetic tree constructions:** Clustal Omega is a multiple sequence alignment program for protein sequences and the alignment is then used to generate a phylogenetic analysis and the final phylogenetic tree is displayed. In the study the Clustal Omega was used to obtain phylogenetic analyses of 6 casein protein sequences in mammals [12].

**Secondary structure prediction:** The GOR IV at EXPASY, YASPIN of Centre for Integrative Bioinformatics VU and PressAPro at Laboratory of Bioinformatics and Computational Biology of Institute of Food Science, CNR, Italy were used to predict secondary structures of casein alpha S1 sequences [13-15]. 

**Physico-chemical properties:** The predicted pH value of the isoelectric point (pI), molecular weights and positive and negative charge of proteins were defined by the protparam tool at “SwissProt & TrEMBL” protein database [16]. NetSurfP was used to find out the surface accessible area of all proteins. NetSurfP server provided information about the exposed and buried amino acids of the proteins [17]. 

**Statistical analysis:** Values are presented as the mean ± standard deviation (SD). Analyze of variance followed by LSD test was used to *assess *significance between the test sample and solvent control. *P*<0.05 was considered to be statistically significant. 

## Results

**Figure 1:**
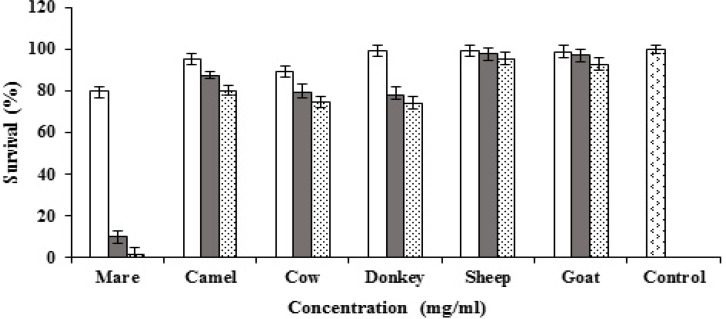
Cytotoxic activities of goat, cow, camel, sheep, mare and donkey milks against MCF7 cells ( 0.5 mg/ml, 1 mg/ml, 2mg/ml

**Figure 2:**
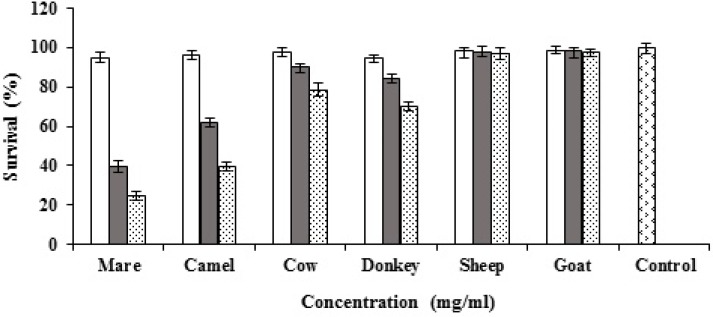
Cytotoxic activities of goat, cow, camel, sheep, mare and donkey caseins against MCF7 cells ( 0.5 mg/ml, 1 mg/ml, 2mg/ml

**Figure 3:**
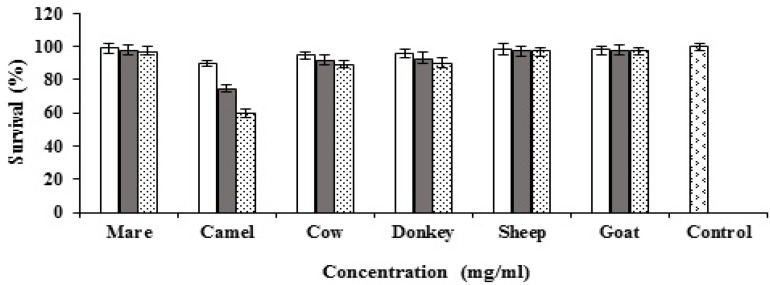
Cytotoxic activities of goat, cow, camel, sheep, mare and donkey whey proteins against MCF7 cells ( 0.5mg/ml, 1mg/ml, 2mg/ml

Goat, cow, camel, sheep, mare and donkey milks and their casein and whey proteins were tested for cytotoxicity against MCF7 cells. All types of milk and the proteins were tested at different concentrations (0.5, 1 and 2 mg/ml). Mare, donkey, cow and camel milks showed dose-dependent cytotoxic activity against MCF7 cells while sheep and goat milks did not reveal any cytotoxic activity (Fig. 1). The highest cytotoxic activity of the caseins was observed by mare milk casein and followed by camel, donkey and cow (Fig. 2). The results of whey proteins demonstrated that camel whey protein showed potent anticancer activity while cow, and donkey milks presented weak anticancer activity (Fig. 3).

**Figure 4:**
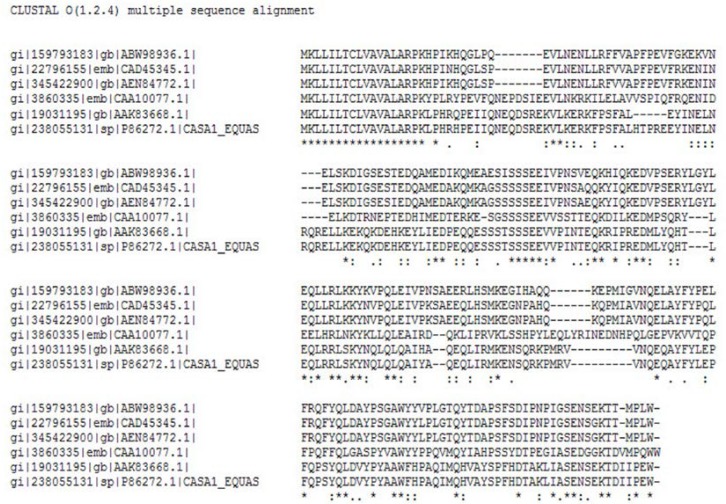
Multiple sequence alignment of casein alpha S1 of goat, cow, camel, sheep, mare and donkey

A multiple sequence alignment was obtained using Clustal Omega with αs1 casein sequences retrieved from the NCBI database (Fig. 4), also NCBI BLAST results showed 44-89% identity between the casein sequences. The sequence relationships revealed that the αs1casein sequences were not belonged to the highly conserved family.

**Figure 5:**
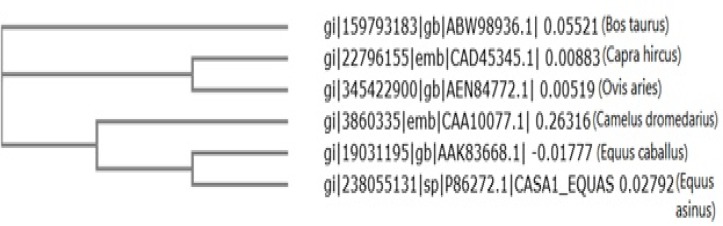
Phylogenetic tree of caseins from different species plotted by CLUSTAL omega.

The phylogenetic tree structure depicts that these proteins were divided into three distinct groups. Members of these two groups were not very diverse and there is a high sequence identity among them. There was no combination of members between these two distinct groups, demonstrating that they were separated early in evolution (Fig. 5).

The results demonstrated that casein types were mostly different in the percentage of alpha helix, beta sheet and random coil. The estimated secondary structure from GorIV, PreSSApro and YASPIN web servers for αs1-casein was 39 to 46% α-helix; 8 to 14% extended strand (β-sheet-like) and 42 to 49% random coil (Table 2). Camel casein had the highest percentage of extended strand, while sheep and goat had the lowest percentage in this regard. The maximum percentage of α-helix was obtained by horse and donkey caseins and the minimum percentage was also obtained by goat and camel caseins.

**Table 2:** **:** Secondary structure prediction of casein alpha s1 by GORIV, PreSSApro and YASPIN

**αS1 Casein**	**α helix (%)**	**Extended strand (%)**	**Random coil (%)**
Horse	46.85	10.21	42.92
Camel	39.33	14.11	46.54
Cow	42.59	10.74	45.32
Goat	42.05	8.72	49.21
Sheep	42.46	9.19	48.28
Donkey	44.85	10.29	45.46

The protparam results of Physico-chemical properties are showed in Table 3. The aliphatic index of camel and cow casein proteins were clearly higher than other casein types. The results also demonstrated that positive and negative charges were clearly different between these casein types. Horse and donkey caseins had the highest positive charge and followed by camel, cow, sheep and goat caseins. The highest negative charge of caseins was obtained by camel casein and followed by donkey, horse, cow, sheep and goat caseins. The results of buried and exposed residues were also attained by NetsurfP (Table 4). The maximum and minimum percentages of buried residues were attained by cow and horse caseins respectively. Conversely, horse and cow caseins showed the highest and lowest percentages of exposed residues respectively.

**Table 3:** Physico-chemical propertie of casein alpha S1 by protparam

**αS1 Casein**	**Mw**	**Neg charge**	**Pos charge**	**Aliphatic index**
Horse	25.30	32	28	82.83
Camel	25.84	36	25	85.14
Cow	24.53	32	21	85.19
Goat	24.27	26	20	82.06
Sheep	24.30	27	21	83.88
Donkey	25.96	33	29	80.92

**Table 4:** Percentage of buried and exposed residues of casein alpha s1 by NetsurfP

**αS1 Casein**	**Buried%**	**Exposed%**
Horse	40.09%	59.90%
Camel	41.89%	58.10%
Cow	45.79%	54.20%
Goat	45.32%	54.67%
Sheep	44.39%	55.60%
Donkey	44.23%	55.76%

## DISCUSSION

Milks contain more than 25 different proteins. Approximately 82% of mammalian milk proteins are caseins, and the remaining 18% is whey proteins. In this study, anti-proliferative activity of different milks, caseins and whey proteins were tested. The results demonstrated that mare, donkey and cow milks and their caseins have potent anticancer activity against MCF7 cells. The highest anticancer activity was obtained by mare casein and followed by camel, donkey and cow caseins. The results showed that sheep and goat milks and their caseins did not have any anticancer activity. These evidences suggested that anticancer activity of animal milks was mostly related to the physical and chemical characteristics of their caseins. Several results have been reported about anticancer activity of animal milks. The cytotoxic activity of mare^,^s milk against Raji and CEM-SS cells has been also reported previously [18]. One study about the cytotoxic activity of a peptide derived from human αs1 casein (αs1-casomorphin and αs1-casomorphin amid) against T47D cells was reported [7]. Our results demonstrated that whey proteins of camel, cow and donkey have week cytotoxic activity. Similar studies reported that donkey and bovine whey proteins have cytotoxic activity against A549 and human breast cancer cells [6]. In the present study as1 casein sequences of cow, goat, sheep, mare, donkey and camel were analyzed using bioinformatics method. *In silico* results showed that, the highest percentage of negative and positive charges achieved by mare, camel and donkey caseins. A similar pattern was also obtained for alpha helix structure of the caseins as mare casein had the highest level of alpha helix structure and potent anticancer activity.

This study suggests that there is a striking correlation between anti-cancer activity of milk caseins and their physicochemical properties such as alpha helix structure and positive and negative charges. Huang et al showed highly charged COS (chitooligosaccharide) derivatives could significantly reduce cancer cell viability, regardless of the positive or negative charges [19]. The secondary structure (α-helical or β-sheet) of anticancer peptides is related to the high percentage of negative and positive charges. Several studies reported that alpha helical cationic anticancer peptides (ACPs) displayed unique mechanisms of action and several extraordinary properties such as broad spectrum activity and rapid action and cancer cells couldn’t fight against them [20]. According to our result, milk casein may be a good candidate for in vivo treatment of cancer patients.
